# GAAPO: genetic algorithmic applied to prompt optimization

**DOI:** 10.3389/frai.2025.1613007

**Published:** 2025-09-22

**Authors:** Xavier Sécheresse, Jacques-Yves Guilbert–Ly, Antoine Villedieu de Torcy

**Affiliations:** Biolevate, Paris, France

**Keywords:** artificial intelligence, prompt engineering, genetic algorithmic, LLM, prompt optimization

## Abstract

Large Language Models (LLMs) have demonstrated remarkable capabilities across various tasks, with their performance heavily dependent on the quality of input prompts. While prompt engineering has proven effective, it typically relies on manual adjustments, making it time-consuming and potentially suboptimal. This paper introduces GAAPO (Genetic Algorithm Applied to Prompt Optimization), a novel hybrid optimization framework that leverages genetic algorithm principles to evolve prompts through successive generations. Unlike traditional genetic approaches that rely solely on mutation and crossover operations, GAAPO integrates multiple specialized prompt generation strategies within its evolutionary framework. Through extensive experimentation on diverse datasets including ETHOS, MMLU-Pro, and GPQA, our analysis reveals several important points for the future development of automatic prompt optimization methods: importance of the tradeoff between the population size and the number of generations, effect of selection methods on stability results, capacity of different LLMs and especially reasoning models to be able to automatically generate prompts from similar queries… Moreover, we decided to use limited size datasets extracted from the original databases to ensure real life applications of our prompt optimization strategy. Finally, we provide insights into the relative effectiveness of different prompt generation strategies and their evolution across optimization phases. These findings contribute to both the theoretical understanding of prompt optimization and practical applications in improving LLM performance.

## 1 Introduction

Large Language Models (LLMs) have gained significant attention following the public release of generative AI assistants such as ChatGPT (2022) and Claude (2023). A critical factor in maximizing these models' effectiveness lies in the quality of input prompts (Sahoo et al., 2025) - the instructions that guide LLMs toward generating relevant outputs. While the impact of prompting on LLM performance has been well-documented through various benchmarks, the process typically relies on manual adjustments, making it both time-consuming and prone to human error (Schulhoff et al., 2024). This highlights the necessity for developing automated methods to fully harness the capabilities of modern LLMs.

In response to this need, several machine learning approaches have been developed to automate prompt optimization. Reinforcement learning has been employed to optimize evaluation costs and computational efficiency (Yang et al., 2024; Ma et al., 2023), while in-context learning focuses on improving prompt performance through example-based learning (Dong et al., 2024). Regression techniques have been explored to establish direct relationships between prompt characteristics and model performance (Feffer et al., 2024). These diverse approaches aim to streamline the prompting process, reducing the reliance on manual intervention while addressing different aspects of prompt optimization.

Recent research has shown that smaller language models can achieve performance comparable to larger LLMs through various optimization techniques such as distillation (Xu et al., 2024) and prompt engineering (Schulhoff et al., 2024). While traditional approaches like distillation modify model weights, prompt optimization offers a more flexible alternative: it enhances model performance without altering the underlying architecture. This approach is particularly valuable as it can be applied to any LLM regardless of size or architecture, providing a generalizable framework for task-specific optimization while maintaining cost-effectiveness.

In this work, we introduce GAAPO (Genetic Algorithmic Applied to Prompt Optimization), an algorithm that integrates different prompt generation strategies into a hybrid prompt optimizer. This innovative approach capitalizes on the strengths of diverse techniques, ensuring optimal performance. Crucially, it maintains a detailed record of the evolution of prompting strategies, which is essential for tracking progress and making informed adjustments. The design of this optimizer prioritizes adaptability, ensuring it can seamlessly incorporate future advancements in the field, thereby remaining relevant and effective as new techniques and models emerge.

## 2 Materials and methods

### 2.1 Related works

#### 2.1.1 Prompt engineering

Prompt engineering is a critical aspect of working with large language models (LLMs), as it involves crafting inputs that guide the model to produce desired outputs. It has been demonstrated that this step is critical to enhance LLM capabilities (Schulhoff et al., 2024). However, this process requires a deep understanding of both the model's capabilities and the specific task at hand. Traditionally, prompt engineering has been a manual process (Bsharat et al., 2023), relying on human intuition and expertise to iteratively refine prompts for optimal performance.

#### 2.1.2 Automatic prompt engineering

The limitations of manual prompt engineering have led to the development of automated approaches. These methods utilize machine learning algorithms to automatically generate and optimize prompts, reducing the need for manual intervention and democratizing access to advanced language processing capabilities. To standardize these developments, frameworks like DSPy (Khattab et al., 2023) have emerged, providing a systematic approach to developing and evaluating automatic prompt optimization methods. Various approaches have been explored in this field, from “gradient-oriented” prompt evolution (Shin et al., 2020) to more sophisticated optimization techniques. Notable advances include APO (Pryzant et al., 2023), which introduced gradient-based prompt optimization, while OPRO (Yang et al., 2024) demonstrated the effectiveness of using LLMs themselves as optimizers. These automated methods can efficiently explore vast prompt spaces, identifying optimal prompts that maximize model performance on specific tasks. This systematic approach has become increasingly important as LLMs are deployed in diverse applications, where task-specific prompt optimization can significantly impact performance.

**Figure 1:**
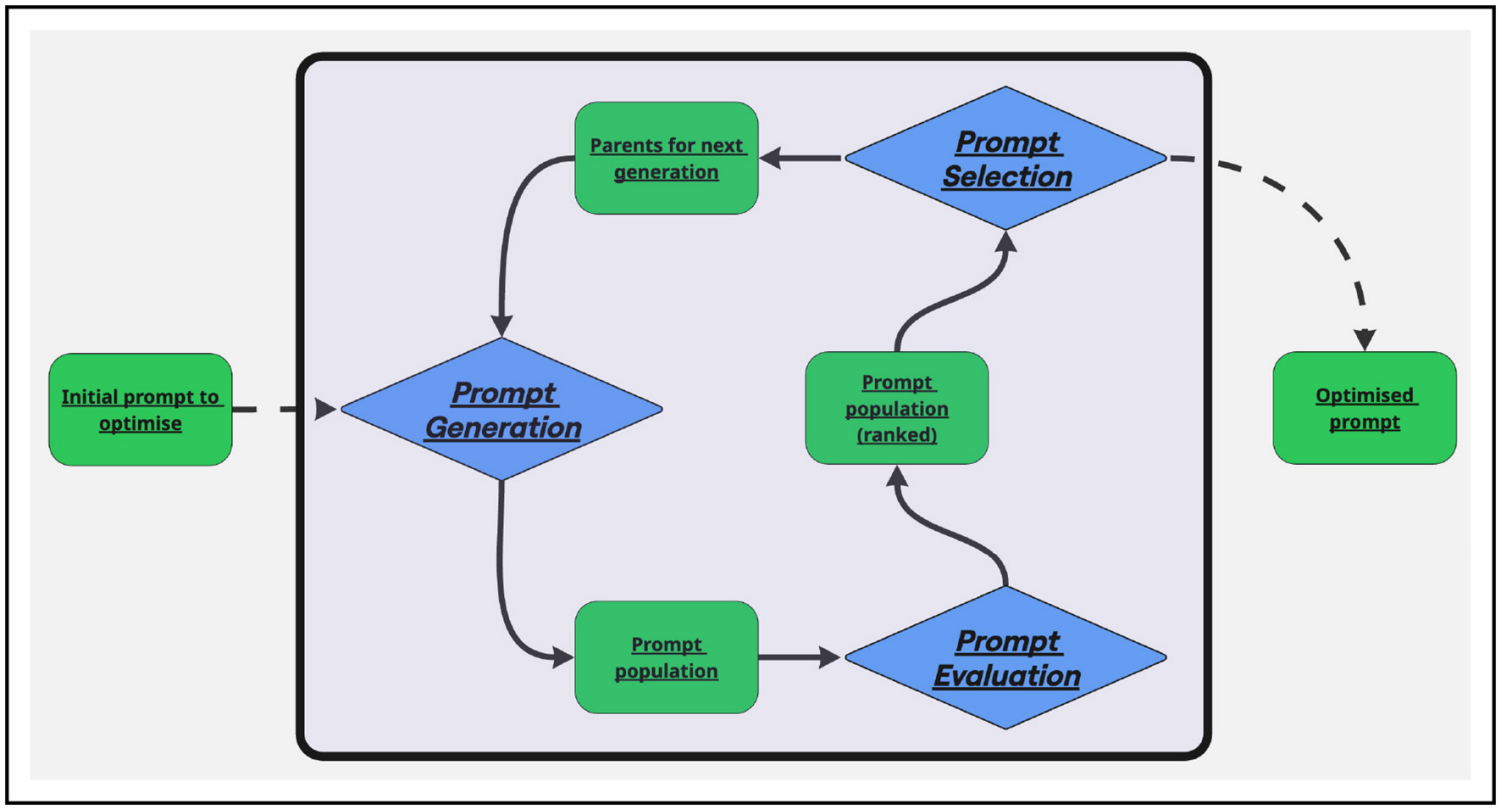
Schema of the general automatic prompt optimization process.

Most prompt optimization techniques follow the same architecture, described in the Figure 1.

#### 2.1.3 Genetic algorithm

Genetic algorithms (GAs) are a class of optimization techniques inspired by the principles of natural selection and genetics (DeJong, 1988). By mimicking the evolutionary process, GAs have been successfully applied to various machine learning and artificial intelligence tasks (Lambora et al., 2019). They are particularly effective in solving complex optimization problems where traditional methods struggle, thanks to their ability to explore large and poorly understood search spaces. The GA process begins with a randomly initialized population of candidate solutions, each evaluated based on a fitness function that measures its effectiveness in solving the problem. The best-performing individuals are selected for reproduction using evolutionary operators such as crossover, which recombines elements from two solutions, and mutation, which introduces random modifications to enhance diversity.

Over the years, GAs have been widely adopted in nearly every field of machine learning, including feature selection (Yang and Honavar, 1998), neural network optimization (Stanley and Miikkulainen, 2002), hyperparameter tuning (Ganapathy, 2020), clustering (Maulik and Bandyopadhyay, 2000), and reinforcement learning (Whiteson and Stone, 2006). For example, GAs have been used to optimize neural network architectures by evolving network topologies and weight configurations, improving model performance and efficiency (Stanley and Miikkulainen, 2002). In reinforcement learning, they have been leveraged to evolve policies and reward functions, enabling agents to learn complex behaviors (Whiteson and Stone, 2006). Additionally, hybrid approaches combining GAs with local search techniques have been developed to improve convergence speed and accuracy (Merz and Freisleben, 2000). Parallel implementations of GAs further enhance their scalability, allowing them to tackle large-scale optimization problems efficiently (Cantú-Paz, 2001). The adaptability and robustness of genetic algorithms make them a powerful tool for advancing machine learning methodologies and solving a diverse range of computational challenges.

#### 2.1.4 Application to prompt optimization

Genetic algorithms have been previously explored in prompt optimization, though their implementations often focus on specific aspects of the prompt space. EvoPrompt (Guo et al., 2023) introduces a basic evolutionary approach where new prompts are primarily generated through crossover operations, combining successful segments from parent prompts followed by linguistic refinement. This method, while effective, primarily explores structural variations within a limited scope of the prompt space. A more sophisticated approach is demonstrated by PhaseEvo (Cui W. et al., 2024), which implements a two-phase evolutionary strategy. The first phase employs global mutations to identify promising regions in the prompt space, effectively searching for potential global optima. The second phase then applies more focused optimizations through semantic mutations and gradient-based refinements.

However, despite their innovative contributions, these approaches operate within relatively narrow paradigms of prompt generation and modification. While they effectively handle structural and semantic modifications, they don't fully explore the broader spectrum of prompt transformation strategies. Moreover, they lack a comprehensive framework that could integrate existing prompt optimization techniques or adapt to emerging methodologies in the field. This limitation in extensibility and modularity restricts their ability to evolve alongside new developments in prompt engineering.

### 2.2 Datasets

**ETHOS dataset**

The ETHOS (Ethics in Text - Hate and Offensive Speech) multilabel dataset (Mollas et al., 2022) is a specialized benchmark designed to evaluate hate speech recognition capabilities in language models. It consists of 443 carefully annotated text samples categorized across eight distinct dimensions of hate speech and offensive content, including race, gender, and violence. Each sample in the dataset is labeled to indicate the presence or absence of specific types of harmful content, enabling fine-grained evaluation of model performance in detecting various forms of hate speech. The dataset's multi-label structure allows for comprehensive assessment of language models' ability to identify intersecting forms of discriminatory or offensive content, making it particularly valuable for evaluating ethical content moderation capabilities. The balanced distribution across different categories of hate speech ensures robust evaluation across the spectrum of harmful content typically encountered in real-world applications.

**Note:** This dataset is different than the standard ETHOS dataset used in several papers of prompt optimization which correspond to the detection of messages as hate speeches and not their classification into different subcategories of hate speeches.

**Complementary datasets**

To assess performances of our approach on a wide range of tasks, we evaluated our model on 3 other datasets, alongside with ETHOS-multilabel:

- The MMLU-Pro (Massive Multitask Language Understanding Professional) (Wang et al., 2024) dataset extends the famous MMLU (Hendrycks et al., 2020) dataset by complexifying it to a professional level, with our focus on two key subcategories. The Engineering subcategory evaluates technical understanding across various engineering disciplines, testing knowledge of fundamental principles, technical specifications, and complex problem-solving approaches encountered in professional practice. The Business subcategory assesses comprehension of management principles, corporate strategy, financial decision-making, and organizational behavior through practical business scenarios.
- GPQA (General Physics Question Answering) presents a specialized evaluation framework for physics understanding through multiple-choice questions. The dataset covers a broad spectrum of physics topics, from mechanics to quantum physics, requiring both theoretical knowledge and practical problem-solving abilities. Questions are designed to test not only recall of physical principles but also their application in solving concrete problems, making it an effective benchmark for assessing scientific reasoning capabilities in LLMs (Rein et al., 2024).

### 2.3 Methods

#### 2.3.1 Models

##### 2.3.1.1 GAAPO: genetic algorithmic applied to prompt optimization

**Figure 2:**
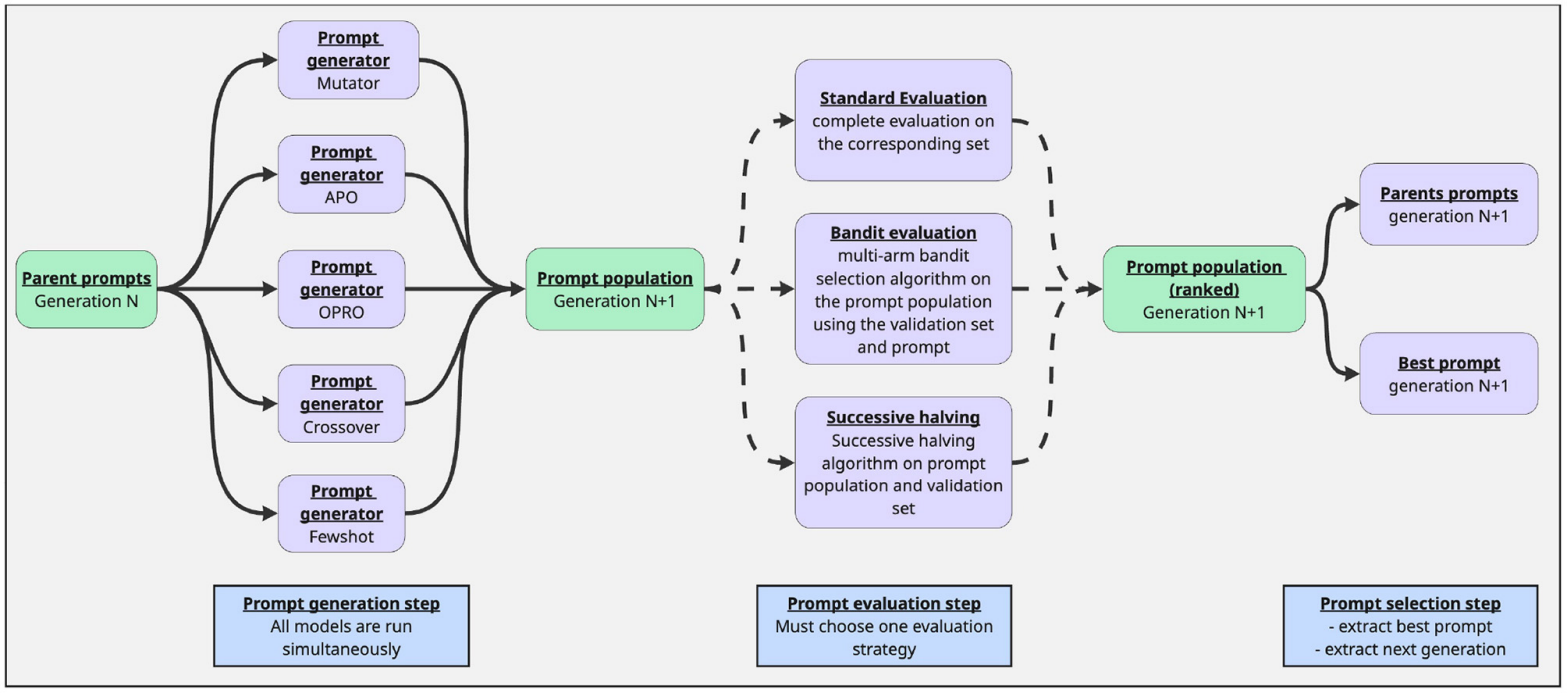
Description of the GAAPO optimization process.

GAAPO follows the principles of genetic algorithms to evolve and optimize prompts through successive generations. The algorithm combines multiple prompt optimization strategies to explore a broader prompt space than previous methods, leveraging the strengths of each approach while maintaining the evolutionary nature of genetic algorithms. The optimization pipeline, inspired by existing works (Pryzant et al., 2023; Cui A. et al., 2024) and described in the Figure 2, operates in three distinct phases during each generation:

- Generation phase: New prompt candidates are created using multiple strategies, with each strategy operating on a subset of high-performing prompts from the previous generations.
- Evaluation phase: The newly generated population is evaluated on the validation set using either exhaustive evaluation or a bandit-based approach to optimize computational resources.
- Selection phase: Top-performing prompts are selected based on their evaluation scores to serve as parents for the next generation, ensuring best performers are used as parents at all time for the future generations.

This iterative process combines the exploration capabilities of genetic algorithms with specialized prompt optimization techniques, enabling efficient navigation of the prompt space while maintaining diversity in the population.

**Prompt generation**

The genetic algorithm framework incorporates multiple prompt generation methods, each implementing distinct optimization strategies as detailed in Sections 2.3.1.2.1, 2.3.1.2.2. These methods, summarized in Table 1, represent diverse approaches to prompt optimization, each with its own strengths and limitations. The hybrid nature of GAAPO leverages this diversity by combining these complementary strategies within a single optimization framework. This integration enables the algorithm to capitalize on the advantages of each method while mitigating their individual limitations through iterative application of varied optimization approaches. The synergistic combination of these methods allows for more comprehensive exploration of the prompt space than would be possible with any single strategy. Examples of generated prompts on a real-world task are presented below.

**Table 1:** Comparison of prompt generation methods in hybrid genetic optimizer.

**Method**	**Advantages**	**Drawbacks**
Mutations	• Simple and efficient implementation • Multiple mutation strategies available • Maintains prompt diversity • Low computational cost	• Can produce invalid prompts • Changes might be too random • Limited by predefined mutation strategies
APO	• Error-driven optimization • Targeted improvements based on failure analysis • Systematic approach to prompt refinement	• Computationally expensive • Requires error examples • May overfit to specific error patterns
OPRO	• Learns from successful prompts • Efficient use of historical information	• Dependent on quality of previous generations • Can converge to local optima • Higher LLM usage per generation
Crossover	• Combines successful prompt features • Preserves effective components • Low computational cost	• Simple splitting might break prompt coherence • Requires multiple good parents • Can produce semantically invalid combinations
FewShot	• Improves prompt with concrete examples • Helps model understand edge cases • Direct performance feedback	• Can make prompts too lengthy • Risk of overfitting to examples • Limited by example quality and availability

To streamline the optimization process, we unified the selection and evaluation phases across all different optimization methods into a single coherent framework. This architectural decision maintains only the generative (expansion) phases of these algorithms, integrating them as candidate generation strategies within GAAPO's evolutionary cycle. This simplification allows for consistent evaluation metrics and selection criteria across all generated candidates while preserving the unique prompt generation characteristics of each method.

Compared to already existing GA-related prompt optimization methods, this framework allows a wider exploration of the prompt space, leveraging advantages of all implemented methods and not focusing on single-algorithm local improvements.

**Evaluation**

To meaningfully compare new prompts, we evaluate them on a subset of the task we have at hand and compare their accuracy (in the current setting).

Several strategies has been implemented for the evaluation process to rank the individuals in each generation:

- Complete evaluation: Run a standard evaluation of each prompt on the evaluation set and rank new prompts according to their accuracy.
- Successive halving (SH) process (Schmucker et al., 2021): prompt accuracies are compared on a subset of the dataset, the top-performing half of the models is retained, and the survivors are evaluated on a new subset. This process is repeated iteratively until only a few models remain. This approach allows to drastically reduce the number of API calls but increases the risk to remove interesting prompts from the evaluation very early due to the disparity of evaluations results on subsets.
- Bandit selection algorithm (Slivkins, 2024): run a multi-arm selection bandit algorithm. Evaluate subsets of the prompt population on batches of data, and apply the UCB-E reward model (Han et al., 2024) to identify the best arms. Note that this method was also used in the original paper of APO (Pryzant et al., 2023).

**Selection**

The selection step used to generate the new parents at each generation is quite simple. We simply chose, among all the prompts which have been evaluated, the best according to their evaluation score.

##### 2.3.1.2 Generation methods

This section describes the prompt optimization methods incorporated into our study, which function as constituent elements of GAAPO and, for some of them, as comparative baselines for the evaluations.

Next subsections present the details of the models along with prompts obtained at the end of the optimization process (for baselines) for illustrative purpose. The task was to optimize the following prompt to better detect hate speech classes, using the ETHOS-multilabel dataset (presented in Section 2.2):

A message from a user, your goal is to determine if this message is a hate speech or not and in case it is, classify it: user_message. Possible class for the hate speech are: violence, directed_vs_generalized, gender, race, national_origin, disability, religion, and sexual_orientation.

###### 2.3.1.2.1 “Forced” evolutions

The first categories of generators were directly inspired from standard prompt optimization models, described below. This methods directly use previous prompts to generate new ones, by using the errors made (APO) or trying to expand a prompt trajectory (OPRO), hence the “forced” evolution.

**OPRO: Optimization by PROmpting** Optimization by PROmpting (OPRO) (Yang et al., 2024) is an iterative prompt optimization algorithm that leverages large language models to generate and refine prompts through a trajectory-based optimization approach. The algorithm maintains a trajectory of the top-performing prompts, ranked by their performance scores, and uses this historical information to guide the generation of new candidates. During training, OPRO employs a stochastic dropout mechanism on the trajectory of best-performing prompts to maintain diversity and prevent convergence to local optima. The filtered trajectory then serves as input for the generation of new candidate prompts, which are subsequently evaluated on the current set. This evaluation process updates the trajectory, maintaining a dynamic optimization path. Example of obtained prompt:

Analyze the message to determine if it is hate speech: user_message.
Consider the following factors in your analysis:
- Presence of derogatory language or slurs targeting race, gender, religion, or other protected characteristics.
- Any explicit or implicit threats, calls to violence, or incitement of harm.
- Discriminatory statements that demean or degrade individuals or groups.
- The overall tone, intent, and context of the message.
Ensure your reasoning is thorough and your solution is actionable and clear. Possible categories for hate speeches are: violence, directed_vs_generalized, gender, race, national_origin, disability, religion, and sexual_orientation.

**ProTeGi: Prompt Optimization with Textual Gradients**

**Figure 3:**
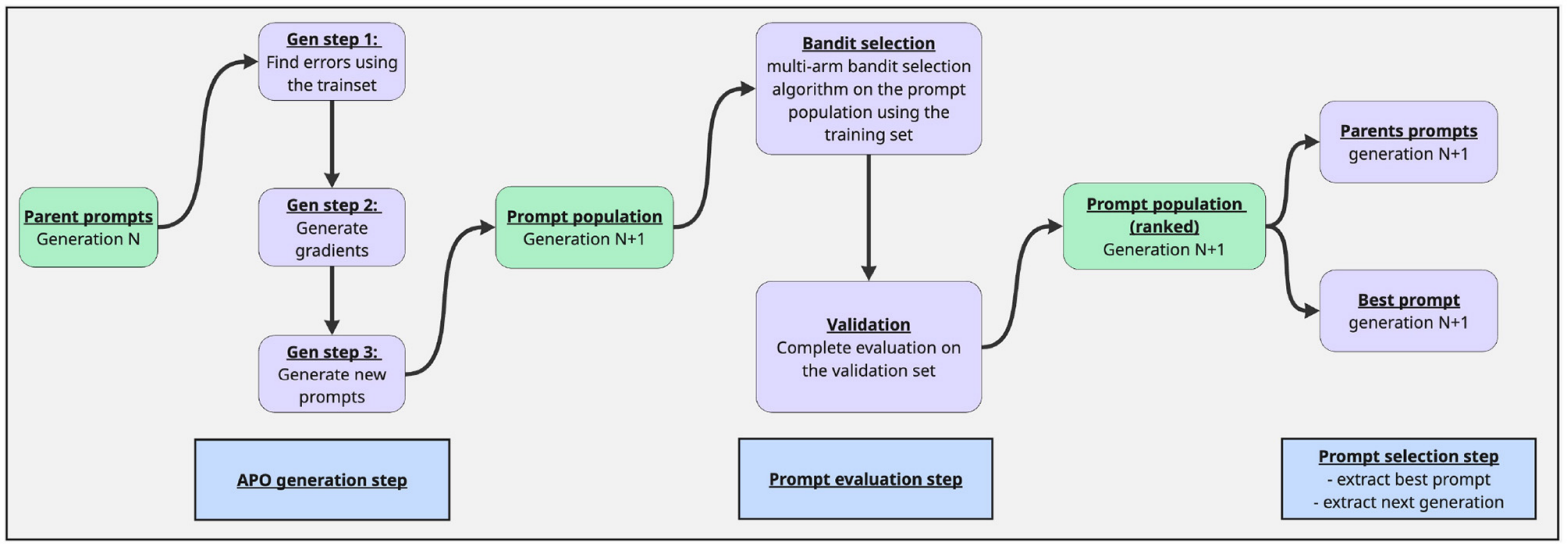
Description of the APO optimisation process, which served as a basis for GAAPO.

The Automatic Prompt Optimizer (APO/ProTeGi) (Pryzant et al., 2023) is an iterative algorithm designed to automatically optimize prompts for Large Language Models through a three-phase process described in Figure 3. The expansion phase begins by evaluating existing prompts to identify errors, which are then grouped for focused analysis. The algorithm generates improvement “gradients” from these errors and creates new candidate prompts. In the selection phase, APO employs multi-armed bandit strategies [such as epsilon-greedy (Kuleshov and Precup, 2014) or Bayesian UCB (Han et al., 2024)] to efficiently identify promising candidates. This approach balances the exploration of new prompt variations with the exploitation of proven patterns, evaluating candidates on small batches for computational efficiency. The validation phase assesses selected candidates on a separate validation set ensuring the robustness of the optimized prompts. Key features include parallel processing, adaptive error analysis, and gradient-guided refinement. Example of obtained prompt:

Analyze the given message to determine if it contains hate speech: user_message.
Definition: Hate speech is defined as language that attacks or diminishes a person or group based on attributes such as race, religion, sexual orientation, gender, disability, national origin, or other protected characteristics. Your task is to:
- Provide a detailed reasoning justifying your classification.
- Categorize the message into the most relevant categories from the following list. Each category should be clearly justified in your reasoning:
+ sexual_orientation: Attacks or offensive language targeting individuals based on their sexual orientation or gender identity.
+ religion: Language that attacks or demeans individuals based on their religious beliefs or affiliation.
+ [… definitions of other categories…]

###### 2.3.1.2.2 Random evolutions

To complement the “forced” evolution optimization strategies, we developed three additional prompt generation methods that were incorporated into GAAPO's framework. These supplementary approaches expand the algorithm's capacity to explore diverse regions of the prompt space. They all use already existing prompts to generate new ones by randomly modifying them.

**Random mutator:** **prompt random mutation**

The Random Mutator serves as a mutation operator within a genetic algorithm framework, designed to explore the vast prompt space through controlled random modifications. This approach draws inspiration from biological mutations in genetic evolution, where random changes can lead to beneficial adaptations. The mutation process operates by randomly selecting from eight distinct mutation strategies, each targeting different aspects of prompt engineering:

- **instruction expansion**: adds detailed guidelines,
- **expert persona injection**: introduces specialized viewpoints,
- **structural variation**: modifies the prompt's architecture,
- **constraint addition**: introduces new boundaries,
- **creative backstory**: weaves narrative elements,
- **task decomposition**: breaks down complex instructions,
- **concise optimization**: streamlines the content,
- **role assignment**: establishes specific model behaviors.

Each mutation creates a new variant of the original prompt, potentially discovering more effective formulations. Like genetic mutations in nature, these modifications can range from subtle adjustments to significant transformations, allowing for both local and global exploration of the prompt space. This random but structured approach enables the discovery of novel prompt variations that might not be obvious through deterministic methods. Example of obtained prompt:

Analyze the message to determine if it is hate speech, using the following structured approach:
- Identify offensive language targeting protected characteristics.
- Assess intent and context.
- Evaluate potential harm.
- Ensure bias-free analysis adhering to hate speech policies.
Provide evidence-based reasoning and actionable solutions. Examples: Racial slurs dehumanize based on race; messages promoting violence incite harm; derogatory gender terms cause marginalization. Analyze: user_message.

**Crossover:** **random prompt merging**

Crossover operations in prompt engineering also draw inspiration from genetic algorithms' recombination mechanisms, but require careful adaptation for text-based prompts. While traditional genetic algorithms can perform straightforward splitting and merging of genetic sequences, prompt crossover needs to maintain semantic coherence and structural integrity. In our implementation, we developed a simple yet effective crossover mechanism: given two parent prompts that have demonstrated good performance, the operation splits each prompt approximately at its midpoint and combines the first half of one prompt with the second half of the other. This approach, while basic (and which could be optimized), provides several advantages:

- It preserves coherent instruction blocks from each parent.
- It enables the combination of different strategic elements (e.g., merging a prompt with strong reasoning guidelines with another that has effective constraint definitions).
- It maintains a balance between exploration and preservation of successful prompt components.

However, this straightforward approach could be enhanced in future work through more sophisticated crossover mechanisms, such as semantic block identification and recombination, or intelligent selection of crossover points based on prompt structure analysis.

Already existing GA-related prompt optimization methods such as EvoPrompt (Guo et al., 2023) are a combination of these first two categories of methods.

**Fewshot:** **in-context learning for prompt optimization**

In-context learning is a fundamental capability of large language models (LLMs) (Dong et al., 2024) that allows them to adapt their behavior based on examples provided within the prompt, without requiring model parameter updates. This ability enables LLMs to understand and emulate patterns from demonstrated examples in real-time. The few-shot algorithm for prompt optimization leverages this capability by augmenting existing prompts with selected examples while maintaining the original prompt's structure and purpose. The process begins by randomly selecting 1 to 3 labeled examples from the training dataset for each parent prompt. These examples are then appended to the original prompt in a structured format, with clear input-output pairs. The algorithm is computationally efficient as it doesn't require complex prompt modifications or extensive evaluations. Instead, it relies on the natural ability of LLMs to learn from examples, making it a practical approach for prompt enhancement while maintaining the original prompt's core functionality.

###### 2.3.1.2.3 GAAPO

Table 1 presents the advantages and drawbacks of methods used in GAAPO. An example of prompt optimized to classify hate speeches is:

You are a team of experts tasked with determining if a given message contains hate speech. Your team consists of three roles:
**The Critic**: Your first responsibility is to carefully analyze the message for any indicators of hate speech, such as offensive language, derogatory terms, or discriminatory content. Consider the context and tone of the message.
**The Problem Solver**: After the critic has provided their analysis, your role is to determine whether the message qualifies as hate speech based on the indicators identified. Consider the broader implications of labeling the message as hate speech.
**The Expert Reviewer**: Your role is to review the analysis and solution provided by the previous roles.
Ensure that the reasoning is thorough, the solution is accurate, and the output is consistent with the examples provided. Here is the message to analyze: user_message.

GAAPO represents a fundamental advancement beyond simple combination of existing optimization strategies by introducing a novel adaptive parameter tuning mechanism and dynamic strategy switching architecture. Unlike traditional approaches that statically combine methods like APO and OPRO, GAAPO employs asophisticated multi-strategy framework that dynamically allocates computational resources across five distinct optimization strategies (mutation, OPRO, APO, few-shot learning, and crossover) based on real-time convergence patterns and performance feedback. The algorithm's key innovation lies in its ability to maintain a “Hall of Fame” of high-performing candidates while simultaneously tracking evolution paths, enabling intelligent strategy selection that adapts to the optimization landscape and possibility to study the best evolutions in order to discover optimization patterns across certain tasks. Furthermore, GAAPO introduces novel evaluation strategies (successive halving, bandit-based selection, and comprehensive evaluation) that significantly reduce computational overhead while maintaining optimization quality as demonstrated in the results. This hybrid architecture not only combines the strengths of existing methods but creates emergent behaviors through strategy interaction, where the combination of multiple approaches produces superior results that cannot be achieved by any single strategy alone.

#### 2.3.2 Optimization framework

Hint Optimization and Prompt Refinement (HOPR) is a Python framework designed for systematic prompt optimization and evaluation. Like DSPy (Khattab et al., 2023), it provides a structured approach to prompt engineering, but with a distinct focus on evolutionary optimization techniques. While DSPy emphasizes the composition and chaining of language model operations through programmatic interfaces, HOPR specializes in automated prompt optimization through a variety of implemented strategies extracted from the state of the art methods for automatic prompt engineering.

The framework is built around modular components: optimizers that implement different prompt generation strategies, metrics for evaluation, and a core system for managing prompt evolution. HOPR's architecture allows researchers to easily implement and compare different prompt optimization techniques, track the evolution of prompts to study the best optimization methods, and maintain a “hall of fame” of top-performing candidates.

Unlike DSPy's focus on prompt composition and application, HOPR emphasizes the development of automatic prompting methods by facilitating the implementation of concurrent strategies on the same problem. While being easily adaptible to new models, this allow a sain and reproducible comparative analysis of different prompt engineering approaches.

A key differentiator is HOPR's hybrid approach, which allows multiple optimization strategies to work in parallel, potentially discovering more effective prompts than single-strategy approaches. This makes it especially valuable for researchers studying prompt optimization methods and practitioners seeking to automatically optimize prompts for specific tasks.

#### 2.3.3 Training pipeline

##### 2.3.3.1 Dataset organization

The optimization process requires careful data partitioning to ensure robust evaluation and prevent overfitting. We divide each dataset into three distinct subsets:

- Training set: used during prompt generation for strategy-specific optimization. APO leverages this set for error analysis and improvement, while the few-shot strategy uses it to select examples for in-context learning.
- Validation set: employed during the optimization process to evaluate and compare generated prompts, enabling the selection of promising candidates for subsequent generations.
- Test set: reserved exclusively for final evaluation, measuring generalization capability and tracking performance evolution across optimization steps.

##### 2.3.3.2 Population management

Each strategy is assigned a weight determining its contribution to the next generation's population. The number of candidates per strategy is calculated by multiplying these weights by the total population size. To maintain the exact desired population size, any remaining slots are allocated to the strategy with the highest weight. This weighted approach ensures:

- Balanced exploration across different optimization techniques.
- Customizable strategy emphasis based on task requirements.
- Consistent population size maintenance throughout generations.

##### 2.3.3.3 Evaluation process

The evaluation of generated prompts follows a systematic approach:

- Initial evaluation on validation set to establish baseline performance.
- Generational evaluation to select promising candidates. Evaluations concerning advocated results in the paper where conducted twice, due to disparities in LLM response and their probabilistic answers construction.
- Final testing on the held-out test set to measure true generalization.

This structured pipeline ensures robust optimization while maintaining the flexibility to adapt to different tasks and requirements through adjustable strategy weights and evaluation parameters.

##### 2.3.3.4 Metrics

**ETHOS multilabel dataset**

For the multi-label classification task of the ETHOS dataset, we employ strict accuracy as our evaluation metric. A prediction is considered correct if and only if the set of predicted labels exactly matches the set of true labels, regardless of their order. Formally, for a sample with true labels *Y* and predicted labels Ŷ, the binary accuracy is defined as:


$$\text{accuracy}(Y,\hat{Y})=\{\begin{array}{ll} 1 & \text{if }Y=\hat{Y}\\ 0 & \text{otherwise} \end{array}$$

where *Y* and *Ŷ* are treated as sets, meaning {*a, b*} = {*b, a*}. The final accuracy score is then computed as the average of these binary evaluations across all samples in the dataset.

**MMLU and GPQA**

For MMLU-Pro and GPQA datasets, we employ standard accuracy as our evaluation metric, where a prediction is considered correct if and only if it matches the correct answer. Formally, for a sample with true answer *y* and predicted answer ŷ, the binary accuracy is defined as:


$$\text{accuracy}(y,\hat{y})=\{\begin{array}{ll} 1 & \text{if }y\equiv \hat{y}\\ 0 & \text{otherwise} \end{array}$$

where ≡ denotes semantic equivalence rather than strict string matching. This equivalence consideration was necessary as these datasets provide multiple-choice answers in a standardized format (typically including punctuation marks like commas), but the LLM sometimes generated correct answers with slight formatting variations. To address this, we implemented an LLM-based evaluation system that validates semantic correctness, ensuring that superficial differences in formatting do not impact the accuracy assessment. The reliability of this approach was verified through manual inspection of a representative sample of model outputs.

**Prompt interpretability and human comparison**

While qualitative analysis of prompts from an end-user perspective might seem valuable, we deliberately eschew direct comparison with human-designed prompts based on empirical evidence from prior research. Studies have demonstrated that human intuition regarding prompt effectiveness often fails to align with actual performance metrics, with human-designed prompts frequently underperforming compared to automatically optimized ones (Zhou et al., 2022). Our approach prioritizes objective performance metrics over subjective human interpretation, as the optimization process explores prompt configurations that may appear counterintuitive to human designers but demonstrate superior empirical performance. This aligns with the broader finding that human prompt design intuition does not reliably predict effectiveness, making qualitative comparisons potentially misleading rather than informative for practitioners.

#### 2.3.4 Experiments

##### 2.3.4.1 Datasets

Three hundred samples were extracted to the orignal ETHOS dataset and separated in 3 subsets: 50 samples for the training set (used in the APO and the fewshot algorithms), 50 samples for the validation set (used for the selection of prompts at each generation) and 200 were used as test set to allow a meaningful comparison of different prompts while limiting the risks of overfitting on other subsets during the optimzation process. Those numbers were chosen as a tradeoff between the budget allowed to the optimization process and the typical size of the datasets which can be obtained in real life optimization tasks. Most results displayed in this paper use the ETHOS dataset.

The statistical significance of our results is ensured through multiple independent trials using the same settings but a different repartition of the selected dataset. This repeated experiments (3 repetitions) were conducted for the direct comparisons of of GAAPO against the selected baselines (result Section 3.1). The stability of results along with the expensive computation costs led us to use this settings only for the first displayed results. For all other experiments, computations were conducted one time with the dataset explained above.

**Models**

We computed prompt optimization for several methods, which we reimplemented, respecting the original description made in their respective papers. In detail, APO (Pryzant et al., 2023), OPRO (Yang et al., 2024) were used as baselines, along with a random mutator described above. In the implementation in GAAPO, no dropout were used for the selection of the prompt trajectory we do not have any necessity to avoid local optimizations issues due to the presence of other prompt generation methods which ensure a more robust exploration of the optimization space. However, when we used OPRO as a baseline, the prompt chosen for the optimization trajectory for the next generation were selected proportionnally to their validation score (a better prompt had a most important probability to be selected as an element of the next trajectory).

In our GAAPO implementation, we've carefully balanced the prompt generation distribution to ensure comprehensive exploration of the optimization space. The distribution is set as follows: random mutations (40%), APO (20%), OPRO (20%), few-shot learning (10%), and crossover operations (10%). For the random mutations component, we implemented an equiprobable selection mechanism among the eight mutation types, with each mutation having an equal probability of being selected. This uniform distribution was chosen deliberately, as we had no prior knowledge about which mutation types would be most effective for different tasks.

These numbers were chosen as a trade-off between random prompt modifications (mutations and crossover), local prompt optimization (APO and OPRO) and in-context learning (fewshot). We deliberately choose to limit the importance of in-context learning as it has already been demonstrated that prompt efficiency scales with the number of given examples. Our goal here is to increase prompt efficiency for very small datasets to have a prompting method which can be used on real life prompts (comparison results are presented in Section 3.1).

However, a complete study of the optimal distribution of ressource allocation across strategies has not been made. Indeed, we believe that this optimisation is task-dependent as the exploration of the prompt optimization space differ between the different tasks.

For each experiment, the number of generations and number of prompt generated at each generation was experimentally determined and will be justified in the Results Section 3.3.

**LLMs**

The experimental setup employs two distinct Large Language Models (LLMs) for different aspects of the optimization process.

For prompt generation, we utilize in most experiments *DeepSeek-R1-distill-LLaMA-70B-versatile*, a state-of-the-art open-source LLM based on the LLaMA architecture. This model, accessed through Groq's inference platform, offers a balance between performance [with state-of-the-art performances on LLM tasks (DeepSeek-AI et al., 2025)] and computational efficiency [with inference times sensibly lower using Groq platform (Abts et al., 2022)]. We compared the performance optimization obtained by this model to others in Section 3.5.

For the target model to be optimized through our prompting process, we employ *GPT-4o-mini* or *llama3-8B-instant* (Grattafiori et al., 2024). We decided to use 2 models to assess the difference in evolution performance (which can be seen in Section 3.2) across different experiment settings, arguing that a prompt optimization could be model dependent.

This configuration allows us to assess the generalizability of our prompt optimization approach while maintaining a clear separation between the prompt generation and evaluation phases of our methodology.

**Generalization**

We evaluated our prompt optimization approach across several widely used datasets, with results presented in Section 3.6. For each dataset, we maintained a consistent splitting strategy: 50 samples for training, 50 for validation, and up to 200 samples for testing (or the maximum available if fewer than 200 samples remained). This standardized approach, first validated on ETHOS, ensures fair comparison across different datasets while maintaining sufficient samples for reliable evaluation. Complementary datasets used for this study are presented in Section 2.2. Note that for GPQA, only 98 samples were used in the testing set.

**Optimization of the selection process**

To optimize the computational budget while maintaining effective prompt selection, we implemented and compared three different selection strategies (see Section 3.7 for results): complete evaluation, successive halving, and bandit selection. These methods present different trade-offs between evaluation accuracy and computational efficiency. For a representative scenario with a test dataset of 50 samples and a population of 50 prompts, the computational requirements vary significantly across methods.

- Complete evaluation, which tests every prompt against every sample, requires 2,500 LLM calls (50prompts × 50samples), while providing exhaustive but computationally intensive evaluation.
- Successive halving (Schmucker et al., 2021) offers a more efficient approach by progressively eliminating underperforming prompts. In our implementation, we evaluate prompts on 20% of the dataset at each iteration and eliminate 40% of the lowest-performing prompts. This process continues until reaching a predetermined number of prompts. This strategy reduces the number of LLM calls to approximately 1,200, representing a 55% reduction in computational cost compared to complete evaluation while maintaining robust selection pressure.
- The bandit selection method (Slivkins, 2024) provides the most efficient tradeoff (Pryzant et al., 2023), evaluating only 20 prompts on 15 samples over 5 iterations. This approach requires approximately 1,500 LLM calls (20prompts × 15samples × 5iterations), achieving a 40% reduction in computational cost compared to complete evaluation. While this method samples less extensively, it leverages statistical efficiency to identify high-performing prompts.

These selection strategies offer different balances between evaluation thoroughness and computational efficiency, allowing practitioners to choose based on their specific constraints and requirements. Our empirical results suggest that both successive halving and bandit selection maintain effective prompt identification while significantly reducing computational overhead.

## 3 Results

### 3.1 Comparison with baselines

**Figure 4:**
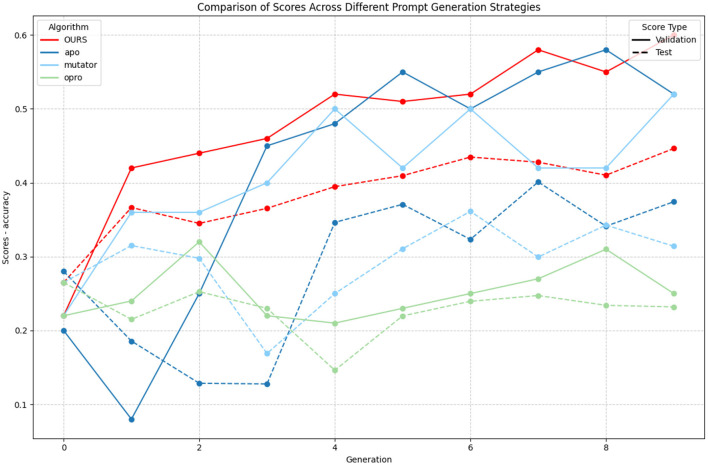
Results obtained by using several prompt generation strategies. LLM-optimizer used: llama-3.1-8B.

The experimental results demonstrate the effectiveness of our proposed GAAPO (Genetic Algorithm Assisted Prompt Optimization) approach on the ETHOS multilabel hate speech classification task. Figure 4 illustrates the validation performance across different prompt optimization strategies over multiple iterations, while Table 2 presents the final test and validation scores. Additionally, obtained prompts are presented in Section 2.3.1.2.

**Table 2:** Test and validations scores for the ETHOS dataset.

**Model**	**Validation score**	**Test score**
Ours	0.60 ± 0.04	0.46 ± 0.02
APO	0.52 ± 0.02	0.38 ± 0.04
OPRO	0.28 ± 0.08	0.24 ± 0.06
Mutator	0.52 ± 0.04	0.34 ± 0.04

Comparison of performance of prompt optimization using llama3-8B-instant (Grattafiori et al., 2024). The bold values correspond to the best scores obtained.

GAAPO demonstrates strong performance on the validation set, achieving a score of 0.46, which significantly surpasses baseline methods including OPRO (0.24), Mutator (0.34), and APO (0.38). The evolution curve in Figure 3 shows GAAPO's ability to maintain consistent improvement throughout the optimization process.

A critical analysis of test and validation scores reveals an important phenomenon common to genetic algorithms: selection bias. This is particularly evident in APO's performance, where results at some iterations highlight a very high difference score between test and validation sets (culminating at 0.3 for the 2nd generation). This extreme disparity illustrates how genetic algorithms can inadvertently optimize for specific test set characteristics rather than general problem-solving capabilities. GAAPO mitigates this selection bias through its diverse strategy portfolio, resulting in more balanced performance between test (0.60) and validation (0.46) scores, suggesting better generalization.

The lower performance of OPRO (test: 0.26, validation: 0.24) indicates that reinforcement learning-based approaches struggle with exploring vast prompt spaces effectively. The Mutator approach achieves intermediate results (test: 0.52, validation: 0.34), but still shows signs of selection bias with its significant test-validation gap. These observations highlight how selection bias can affect different optimization strategies to varying degrees, with GAAPO's hybrid approach providing the most robust defense against this common genetic algorithm limitation.

Moreover, we can see a difference in the original score of the models (at iteration 0, all scores should be identicals). However, due to the disparity in LLM performance and their probabilistic caracters, results are not always exactly consistent across time.

### 3.2 Model evaluation comparison

**Figure 5:**
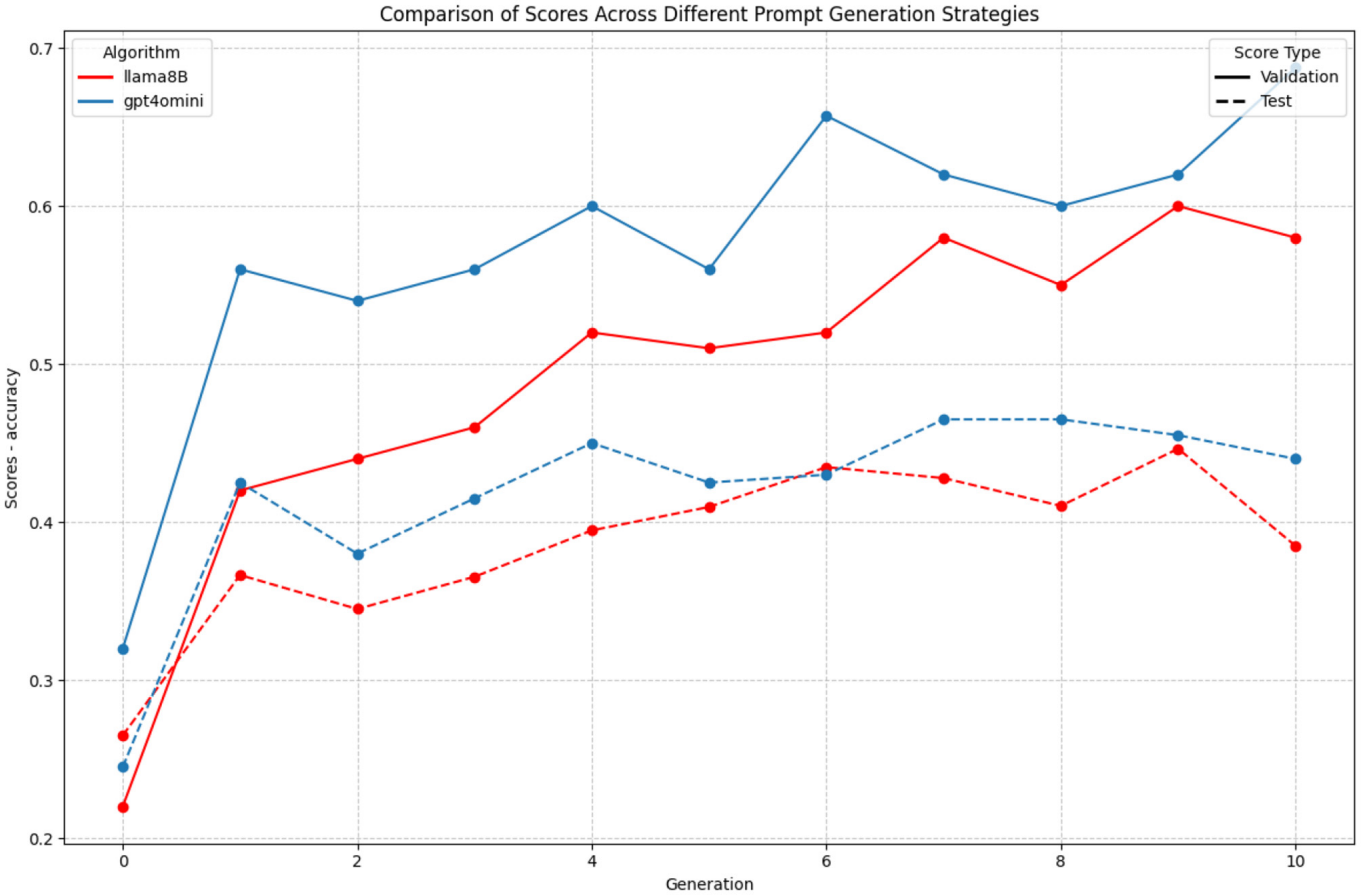
Comparison of optimization trajectories between GPT-4o-mini and LLaMA3-8B models on the ETHOS dataset for GAAPO. The plot shows the evolution of validation scores (solid lines) and test scores (dashed lines) across generations for both models.

Comparing the optimization trajectories between GPT-4o-mini and LLaMA3-8B (displayed in Figure 5) reveals few differences in how these models respond to prompt optimization. Both models show significant improvement from their initial performance, but their learning patterns and final achievements slightly differ.

Both GPT-4o-mini and LLaMA3-8B demonstrate stable optimization trajectories, with consistent learning patterns and similar generalization characteristics across generations. However, GPT-4o-mini achieves notably superior performance, reaching validation scores of up to 0.70 compared to LLaMA3-8B's 0.60. Both models maintain steady optimization paths with comparable stability in their generalization gaps.

Examining test scores reveals GPT-4o-mini's consistent edge in performance, maintaining a 0–0.05 point advantage over LLaMA3-8B throughout the optimization process. However, this superior performance must be interpreted with caution, as both models show signs of potential overfitting in later generations. The increasing gap between validation and test scores after generation 8 suggests that while GPT-4o-mini achieves better absolute performance, careful monitoring of generalization remains crucial for both models.

Given the higher performance metrics of GPT-4o-mini and comparable computational costs between the two models in our experimental setup, we selected GPT-4o-mini as our primary LLM-optimizer for subsequent experiments. This choice was driven by the quantitative advantages in optimization outcomes, while both models demonstrate equally reliable optimization stability.

### 3.3 Influence of population size

We conducted experiments with varying population sizes while maintaining a comparable total number of LLM calls across configurations, as shown in Table 3. The results demonstrate a clear trade-off between population size and the number of generations required. Larger populations (50 prompts) with fewer generations (10) achieve higher test scores (0.68) compared to smaller populations running for more generations (20 prompts, 25 generations, 0.50 test score).

**Table 3:** Test and validations scores for the ETHOS dataset.

**Population size**	**Number of generations**	**Test score**	**Validation score**	**Number of LLM calls**
20	25	0.50	0.42	25,000
30	17	0.56	0.50	25,500
40	13	0.62	0.46	24,500
50	10	0.68	0.46	25,000

Comparison of different population size and number of generations for GPT-4o-mini (Grattafiori et al., 2024).

While the configuration with 30 prompts shows the best validation score (0.50) and a smaller generalization gap, we opted for the 50-10 configuration for several practical advantages. First, larger populations enable better parallelization of prompt evaluation, significantly reducing wall-clock time. Second, this configuration aligns well with optimized selection strategy, which benefits from a larger pool of candidates to select from in each generation.

However, the increased generalization gap in the 50–10 configuration (0.22 points between test and validation scores, compared to 0.08 points for 20–25) suggests a higher risk of overfitting. This observation indicates that while larger populations can explore the prompt space more effectively within fewer generations, they may require more robust validation strategies to ensure generalization. Despite this limitation, the practical benefits of faster convergence and improved parallelization potential make the 50–10 configuration our recommended choice for prompt optimization tasks.

### 3.4 Prompt generators comparison

**Figure 6:**
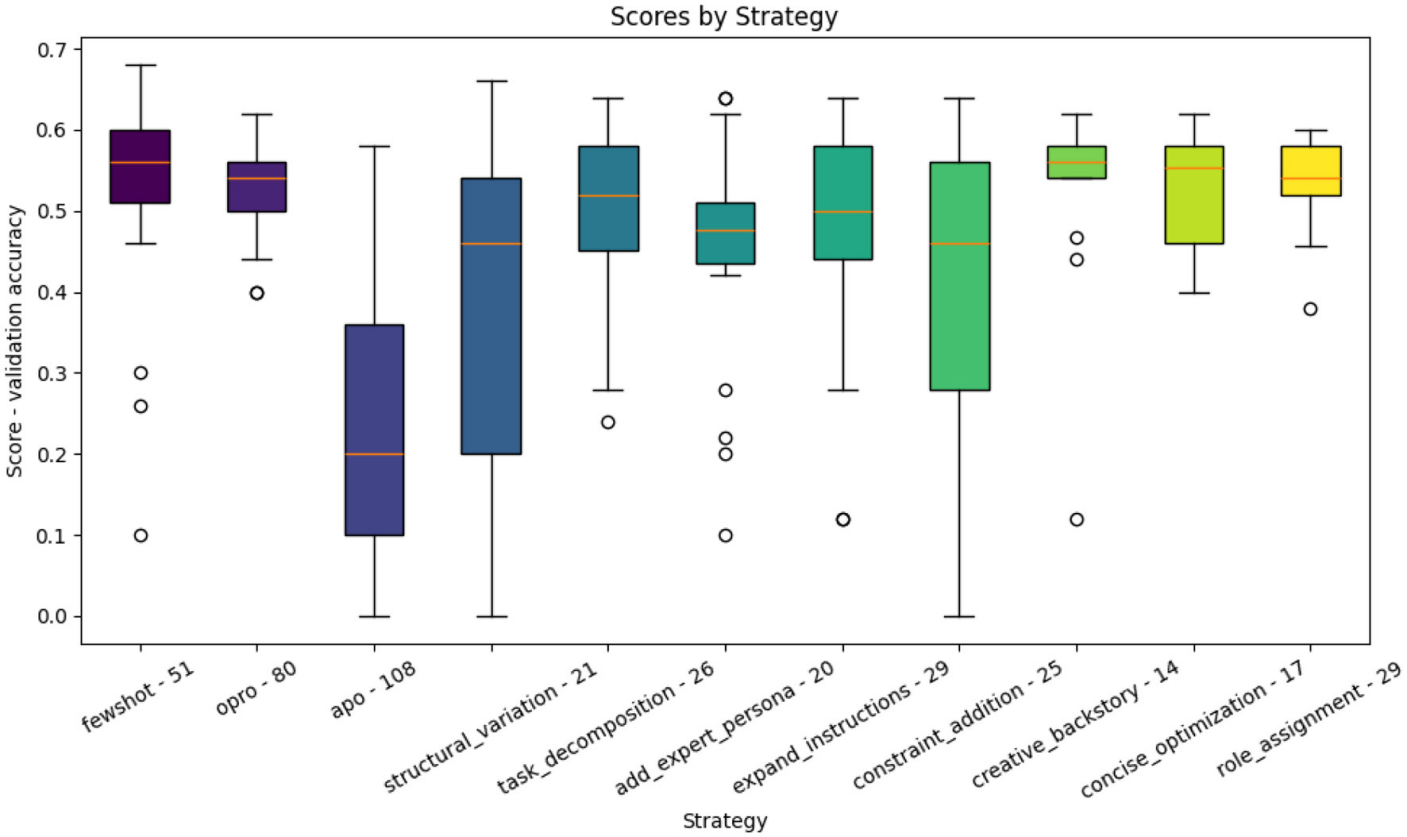
Performance distribution of individual prompt generation strategies in GAAPO on the validation set. Model used: GPT-4o-mini.

**Figure 7:**
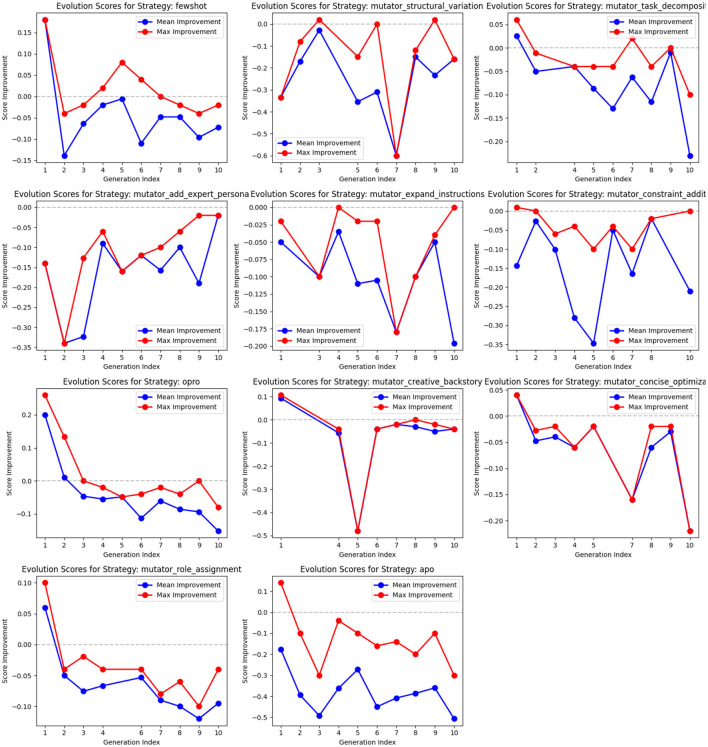
Evolution of improvement scores for each prompt generation strategy across generations. For each strategy, we track both mean improvement (blue) and maximum improvement (red) relative to parent prompts. Mean improvement represents the average score difference between generated prompts and their parents, while maximum improvement shows the best improvement achieved in each generation. Negative values indicate that generated prompts performed worse than their parents. Model used: GPT-4o-mini.

We can now study in detail the prediction made by each prompt generator in GAAPO. We conducted a detailed analysis of each prompt generator's performance in GAAPO through two complementary perspectives. Figure 6 presents the overall distribution of validation scores for each strategy through boxplots, while Figure 7 tracks the improvement potential of each strategy across generations, showing both mean and maximum improvements in score relative to parent prompts.

To obtain these visualizations, we first aggregated all prompts generated by each strategy and analyzed their validation scores (Figure 6). Additionally, we computed the improvement in validation score between each generated prompt and its parent prompt across generations (Figure 7), allowing us to understand not just absolute performance but also each strategy's ability to improve upon existing prompts.

The analysis reveals several key insights about strategy effectiveness and the importance of maintaining diversity in optimization approaches:

- **Strategy effectiveness and stability:** Few-shot learning demonstrates superior performance (median ~0.57) with consistent results, as shown by its compact boxplot and positive improvement scores in early generations. This aligns with existing literature (Dong et al., 2024), highlighting the value of example-based learning. OPRO maintains strong and stable performance (median ~0.55), though its evolution plot shows diminishing improvements over generations. *role_assignment* and *concise_optimization* show reliable performance with tight distributions, but their improvement potential decreases in later generations. It's important to note that while these patterns are observed in this dataset, the relative effectiveness of each strategy can vary significantly across different tasks and datasets, as each optimization problem exists in its own unique optimization space.
- **Evolution patterns:** Most strategies show declining improvement potential over generations, with negative mean improvements in later stages, suggesting they work best in early exploration. APO's boxplot shows high variability (0.10–0.35), but its evolution plot reveals strong initial improvements followed by declining effectiveness, supporting its potential role as an early-stage optimizer. Few-shot learning uniquely maintains positive maximum improvements even in later generations, indicating sustained ability to generate beneficial variations. These patterns demonstrate the complementary nature of different strategies, with each contributing to different stages of the optimization process.
- **Underperforming strategies:** Several mutation strategies, particularly *structural_variation* and *task_decomposition*, consistently show negative improvement scores across generations, suggesting limited effectiveness for the current task. However, completely removing these strategies could be counterproductive for two reasons:

Task dependency: Different tasks may benefit from different prompt modification approaches. What appears ineffective for one task might be crucial for another as every optimization task is learned in a different optimization space. The complementary nature of these strategies means they may excel in different contexts.
Exploration value: Even seemingly underperforming strategies contribute to maintaining genetic diversity, potentially enabling the discovery of novel promising prompt variations through combination with other approaches. This complementary effect is essential for robust optimization. A deeper analysis of the generated population results over time is necessary to assess the noncompliance of a method with a specific task.

- **Strategic implications:** The analysis suggests implementing a dynamic, task-adaptive strategy:

Early generations: Leverage APO and mutation strategies for broad exploration;
Mid-generations: Emphasize few-shot learning and OPRO for stable improvements;
Later generations: Focus on strategies showing consistent positive improvements (few-shot, *role_assignment*) for refinement.
However, it remains fundamental to maintain a minimum weight for all strategies to preserve optimization flexibility across different tasks. The complementary nature of these strategies means that while their relative effectiveness may vary across tasks, their combined presence ensures robust optimization capabilities across diverse optimization spaces.

This comprehensive analysis reinforces the value of GAAPO's adaptable framework, which can accommodate varying strategy effectiveness across different tasks while maintaining the potential benefits of diverse optimization approaches. The framework's ability to dynamically adjust strategy weights while preserving all methods makes it particularly robust for general-purpose prompt optimization across diverse applications.

It should be notice that optimization methods tend to have descending curves which is logical: as we compare new prompts with their parent prompts, the task is more and more difficult (given that the reference prompt improves with the generations). Moreover, studies on other datasets tend to highlight the fact that different prompt optimization methods can perform very differently between tasks, highlighting the importance to keep methods in a general framework and the risk to select optimizers based on their results on a unique dataset.

### 3.5 Model generators comparison

**Figure 8:**
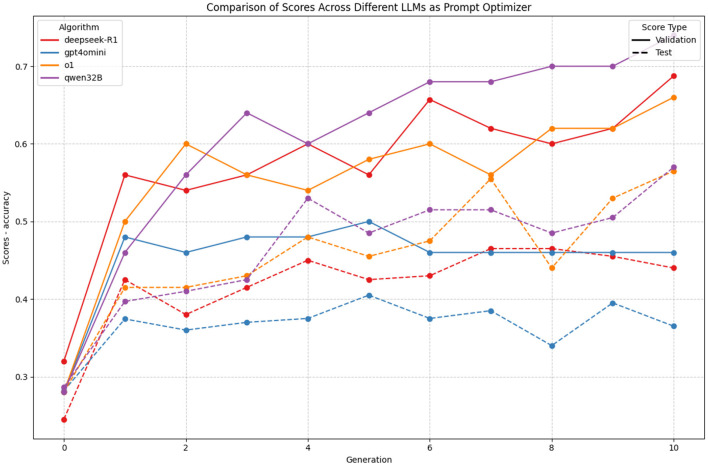
Comparison of different LLMs as prompt optimizers in GAAPO. The plot shows validation (solid lines) and test (dashed lines) scores across generations for four models: QwQ32B, DeepSeek-R1, O1, and GPT-4o-mini. While reasoning-specialized models achieve higher absolute scores, O1 demonstrates better generalization with smaller gaps between validation and test performance.

The comparison of different language models as prompt optimizers reveals striking patterns (which can be seen in Figure 8 in both performance and generalization capabilities. Most notably, reasoning-specialized models (QwQ32B and deepseek-R1) and O1 demonstrate superior performance compared to general-purpose models like GPT-4o-mini.

QwQ32B emerges as the top performer, showing consistent improvement in validation scores from an initial 0.28 to a remarkable 0.70 by generation 10. Its learning trajectory is particularly stable, with steady increases and minimal fluctuations. However, its test scores (dashed line) plateau around 0.55, indicating a significant generalization gap of approximately 0.15 points.

A particularly interesting comparison emerges between DeepSeek-R1 and O1 models. While both achieve strong final validation scores (0.68 and 0.65 respectively), O1 demonstrates notably better generalization characteristics. By generation 10, O1 maintains test scores around 0.55, nearly matching its validation performance, while DeepSeek-R1 shows a larger disparity with test scores around 0.45. This suggests that O1's optimization process, while slightly lower in absolute validation performance, produces more robust and generalizable prompts.

In contrast, GPT-4o-mini shows notably inferior performance. While it achieves quick initial improvement, its validation scores stagnate around 0.45–0.50 after generation 2, with minimal subsequent improvement. However, like O1, it maintains a smaller generalization gap between validation and test scores, suggesting more robust, if modest, optimization capabilities.

The evolution of scores across generations reveals an interesting pattern: while reasoning models continue to improve validation performance until the final generations, o1 maintains a more balanced improvement in both validation and test scores. This suggests that o1 might be particularly valuable for applications where generalization reliability is crucial, even if peak performance is slightly lower than specialized reasoning models.

These findings indicate that while reasoning-specialized models achieve higher absolute performance, o1 offers an attractive compromise between performance and generalization stability, potentially making it more suitable for practical applications where robust generalization is essential.

### 3.6 Applications on other datasets

The experimental results across multiple datasets demonstrate both the effectiveness of our approach and the varying potential for prompt optimization across different tasks. Table 4 presents validation scores for four distinct datasets, revealing several important patterns.

**Table 4:** Validations scores for different datasets.

**Dataset**	**ETHOS multilabel**	**MMLU-Pro engineering**	**MMLU-pro business**	**GPQA**
Initialization	0.28	0.39	0.72	0.38
APO	0.44	0.45	0.73	0.42
OPRO	0.38	0.44	**0.76**	0.43
Mutator	0.40	0.43	0.735	0.43
**OURS**	**0.46**	**0.48**	0.74	0.43

Models used: Deepseek-R1 as Prompt Generator and GPT-4o-mini as Optimizer. The bold values correspond to the best scores obtained.

We can see on Table 4 that our method achieves superior performance on datasets where prompt engineering shows significant potential for improvement. For the ETHOS multilabel classification task, we observe a substantial improvement from the initial score of 0.28 to 0.46, outperforming all baseline methods including APO (0.44), OPRO (0.38), and Mutator (0.40). Similarly, on the MMLU-Pro engineering dataset, our approach reaches 0.48, showing meaningful improvement over the initialization score of 0.39 and competing methods.

However, the results also reveal that not all tasks benefit equally from prompt optimization. The MMLU-Pro Business dataset, with its high initialization score of 0.72, shows minimal room for improvement, with our method and the Mutator achieving only marginal gains (0.73 and 0.735 respectively). This suggests that some tasks may already be well-aligned with LLMs' base capabilities, limiting the potential impact of prompt optimization. The GPQA dataset presents another interesting case where all optimization methods, including ours, achieve similar modest improvements (from 0.38 to 0.43), indicating that some tasks may have inherent complexity barriers that prompt optimization alone cannot overcome.

The varying effectiveness of prompt optimization across tasks can be attributed to multiple underlying factors. First, the overlap between an LLM's training data and the target dataset can create a ceiling effect—if similar examples were present in the training corpus, the model may already demonstrate near-optimal performance with simple prompts. Second, task-specific characteristics such as domain specificity and reasoning complexity influence the optimization potential; technical domains often benefit more from structured prompting than general knowledge tasks. Third, the nature of the required output (e.g., multiple-choice vs. multi-label classification) affects the scope for improvement through prompt engineering. Finally, the fundamental alignment between the task's requirements and the model's learned representations determines whether performance limitations can be addressed through prompt optimization alone or require more substantial interventions such as fine-tuning.

### 3.7 Selection method comparison

We conducted a comparative analysis of the three selection methods on the ETHOS dataset, evaluating their efficiency and performance trade-offs. The computational requirements varied significantly across methods: for a test set of 50 samples, the complete evaluation (“all”) requires 2,500 LLM calls per generation, the bandit approach approximately 1,500 calls, while successive halving (SH) uses only 1,500 calls per generation.

To ensure fair comparison, we also plotted results where the number of calls are equivalent between all methods. We adjusted the test size to 110 samples to obtain the right number of calls for both bandit and SH selection methods.

**Figure 9:**
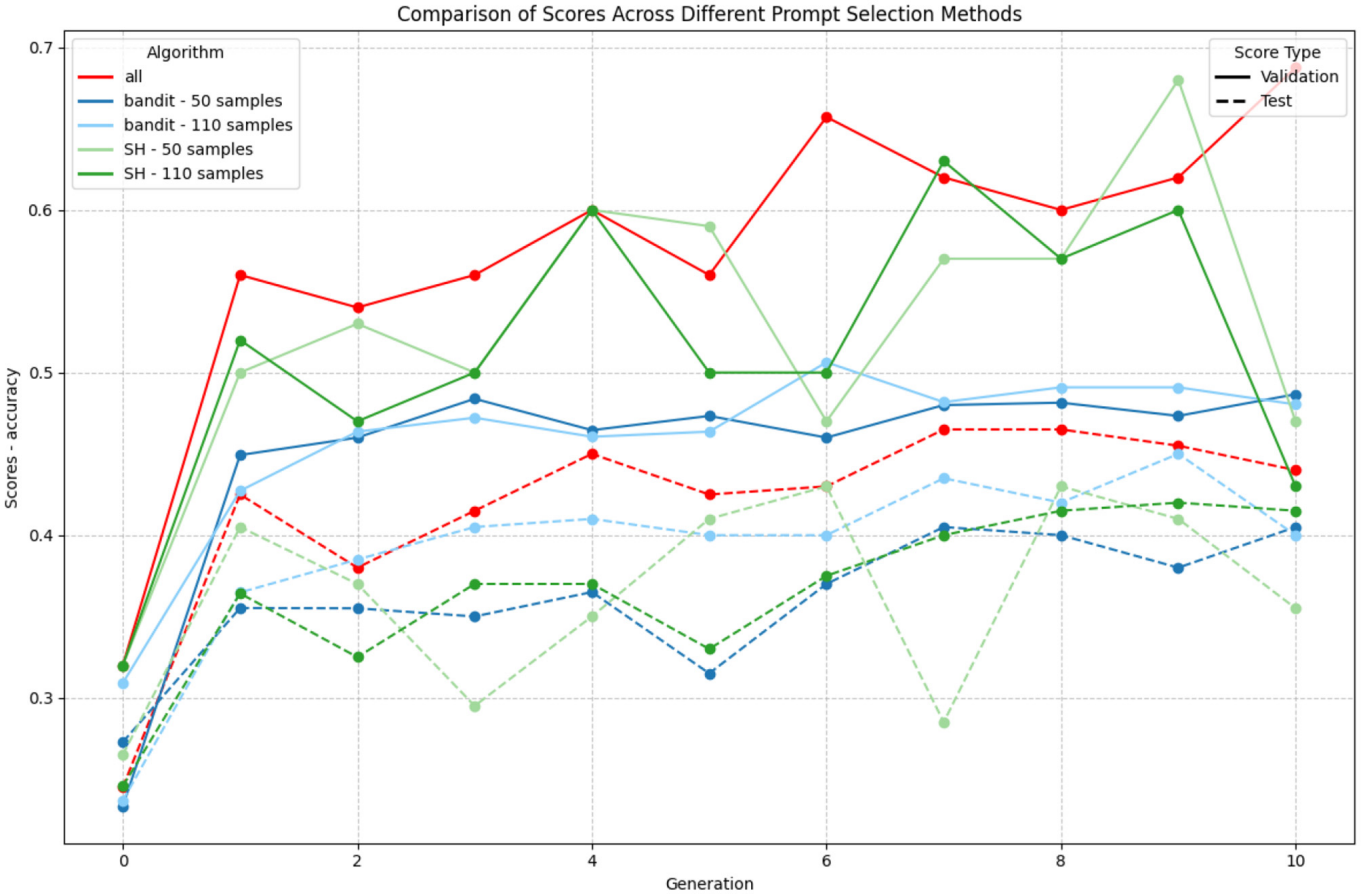
Comparison of different prompt selection strategies during GAAPO optimization. The plot shows the evolution of validation (solid lines) and test (dashed lines) scores across generations for different selection methods. Model used: GPT-4o-mini.

Figure 9 presents the evaluation for both validation and test scores for the 5 mentioned processes: “all,” “bandit” with 50 samples, “bandit” with 110 samples, “SH” with 50 samples and “SH” with 110 samples.

The comparison of different selection strategies reveals compelling insights about the trade-offs between sample size, computational efficiency, and performance stability. The complete evaluation method (“all”), using 50 samples, achieves the highest validation scores (peaking at 0.68) but requires significantly more computational resources. However, our analysis demonstrates that increasing the sample size from 50 to 110 samples for alternative strategies does not necessarily lead to better performance, suggesting that efficient sampling is more crucial than sample size.

The bandit method emerges as particularly noteworthy, showing remarkable stability in both its 50 and 110 sample configurations. Despite using 40% fewer LLM calls, it maintains consistent performance around 0.45–0.50 validation score with minimal fluctuations between generations. More importantly, the bandit approach exhibits a smaller generalization gap between test and validation scores, indicating better resistance to overfitting. However, we can observe a certain drop of performance between this selection method and “all.”

In contrast, successive halving (SH) displays considerable volatility, especially evident in its performance spikes and drops across generations. While SH occasionally matches or exceeds the bandit's performance (reaching peaks around 0.60–0.70), its inconsistency makes it less reliable for practical applications. Interestingly, increasing the sample size for SH from 50 to 110 samples does not significantly mitigate this volatility, nor does it critically improve performances.

These findings suggest that while complete evaluation with 50 samples provides the highest absolute performance, the bandit approach with its reduced computational footprint and stable optimization trajectory offers an interesting alternative. The stability and efficiency of the bandit method, combined with its robust generalization characteristics, make it a choice for resource-conscious prompt optimization scenarios.

## 4 Discussion

### 4.1 Key findings

Our results reveal fundamental aspects of prompt optimization that have significant implications for the field. GAAPO's integration of multiple optimization strategies proves more robust than single-strategy approaches, consistently outperforming baseline methods across different tasks. The framework's ability to leverage diverse techniques while mitigating their individual limitations represents a significant advancement in prompt optimization. This hybrid approach particularly shines in complex tasks where single-strategy methods often struggle to maintain consistent performance.

However, the effectiveness of prompt optimization varies significantly across tasks, with performance improvements ranging from marginal (MMLU-Pro Business: 0.72 to 0.74) to substantial (ETHOS: 0.28 to 0.46). This variability emphasizes the importance of maintaining diverse optimization strategies and suggests that prompt optimization's utility depends heavily on task characteristics and initial model performance. Furthermore, our analysis reveals crucial trade-offs between absolute performance and generalization. While specialized reasoning models achieve higher peak performance, models like O1 demonstrate better generalization characteristics, highlighting the importance of balanced optimization approaches.

From a technical perspective, our study yields several important insights. Population dynamics significantly impact optimization outcomes, with larger populations in fewer generations proving more effective than smaller, longer-running configurations. Selection methods present distinct trade-offs between computational efficiency and performance stability, with bandit-based approaches offering an attractive balance. Additionally, the choice of LLM for prompt generation critically affects both performance and generalization, with different models showing varying aptitudes for prompt optimization tasks.

### 4.2 Limitations

Despite GAAPO's promising results, several limitations warrant discussion.

First, computational constraints limited our ability to conduct comprehensive statistical validation across all experiments. This constraint necessitated the use of a fixed-size dataset and selective statistical analysis, which may introduce unknown biases.

Second, the framework's effectiveness demonstrates task-dependent variability, indicating the need for deeper understanding of task characteristics that influence optimization potential. The observed generalization gaps, particularly in larger population configurations, suggest opportunities for improved validation strategies. Additionally, the computational overhead associated with prompt evaluation remains a significant bottleneck, highlighting the need for more efficient selection methodologies.

Finally, while it would be interesting to evaluate our hybrid optimization framework across all existing NLP tasks to establish comprehensive generalizability, such extensive testing was beyond the scope of our current investigation. Our research objectives focused on developing and validating the hybrid approach specifically for knowledge assessment and reasoning tasks, which represent critical applications in prompt optimization research. Testing across additional domains such as summarization, translation, and text generation would require substantial computational resources and falls outside the scope of this manuscript where our primary goal was to demonstrate the effectiveness of combining multiple optimization strategies.

### 4.3 Future work

Looking forward, several promising research directions emerge. Future work should address the statistical validation limitation through expanded experimental runs to ensure robust statistical validation. Additionally, research should focus on developing adaptive weighting schemes for optimization strategies based on task characteristics and investigating more sophisticated generalization metrics for prompt evaluation. Exploring methods to reduce computational overhead while maintaining optimization effectiveness remains crucial: this could go through a deeper analysis of the algorithms performance in GAAPO to better understand the repartition and evolution of those performances. A complete comparison of those evaluation strategies and how the different optimizations depends on them will be critical to deeply understand optimizations made by models. Moreover, the potential for extending the framework to handle more complex, multi-step reasoning tasks also presents an exciting avenue for future research.

A particularly important direction for future work involves the application domain of hate speech detection. A deeper exploration of ethical concerns, such as potential bias amplification during prompt evolution, is necessary. This investigation would contribute significantly to the responsible development and deployment of prompt optimization techniques in sensitive applications, ensuring that our methods not only improve performance but also uphold ethical standards and promote fairness in AI systems.

While fairness evaluation represents a crucial consideration in hate speech detection systems (and are necessary in order to deeply understand the behavior of LLMs), it falls outside the scope of the current paper, which focuses on prompt optimization methodologies rather than comprehensive fairness assessment. Our study employs the ETHOS multilabel dataset with strict accuracy as the primary evaluation metric, where predictions are considered correct only when the predicted label set exactly matches the true label set. This evaluation framework is designed to assess the effectiveness of prompt optimization techniques in improving classification performance, rather than to evaluate demographic fairness or bias mitigation. An interesting follow-up study and comparison of prompt optimization algorithms could be a comparison of bias evolution through the evaluations of those algorithms. The link between fairness evaluation studies (Bouchard, 2025; Gallegos et al., 2024) and prompt optimization would ensure that performance improvements do not come at the cost of fairness degradation.

Furthermore, the rapid pace of development in prompt optimization research necessitates continuous benchmarking against emerging methodologies. An updated evaluation using more recent benchmarks such as MIPRO (Opsahl-Ong et al., 2024) would provide additional validation of GAAPO's effectiveness and ensure our results remain relevant within the evolving landscape of prompt optimization techniques. This would also enable direct comparison with the latest advances in the field and strengthen the generalizability of our findings.

## 5 Conclusion

Genetic Algorithm Applied to Prompt Optimization (GAAPO) represents a significant step forward in automated prompt optimization, offering a flexible, robust framework that can adapt to various tasks while maintaining strong performance characteristics. The framework's ability to combine multiple optimization strategies while managing their individual limitations provides a promising direction for future developments in prompt engineering. As language models continue to evolve, frameworks like GAAPO will become increasingly important for efficiently leveraging their capabilities across diverse applications.

Our findings contribute to both the practical implementation of prompt optimization systems and the theoretical understanding of how different strategies interact in hybrid optimization frameworks. This work lays the groundwork for more sophisticated approaches to prompt optimization, potentially leading to more efficient and effective use of language models across a broader range of applications. The demonstrated success of GAAPO's hybrid approach suggests that future developments in prompt optimization should continue to explore the integration of diverse strategies while focusing on maintaining generalization capabilities and computational efficiency.

## Data Availability

Publicly available datasets were analyzed in this study. This data can be found here: https://huggingface.co/datasets/TIGER-Lab/MMLU-Pro, https://huggingface.co/datasets/iamollas/ethos, and https://huggingface.co/datasets/Idavidrein/gpqa/tree/main.
